# Methadone Suppresses Neuronal Function and Maturation in Human Cortical Organoids

**DOI:** 10.3389/fnins.2020.593248

**Published:** 2020-11-23

**Authors:** Wei Wu, Hang Yao, Ila Dwivedi, Priscilla D. Negraes, Helen W. Zhao, Juan Wang, Cleber A. Trujillo, Alysson R. Muotri, Gabriel G. Haddad

**Affiliations:** ^1^Department of Pediatrics, School of Medicine, University of California, San Diego, San Diego, CA, United States; ^2^Department of Cellular and Molecular Medicine, Stem Cell Program, Center for Academic Research and Training in Anthropogeny, Kavli Institute for Brain and Mind, University of California, San Diego, San Diego, CA, United States; ^3^Department of Neurosciences, School of Medicine, University of California, San Diego, San Diego, CA, United States; ^4^Rady Children’s Hospital, San Diego, CA, United States

**Keywords:** methadone, cortical organoid, iPSCs, neural function, ion channel, patch-clamp

## Abstract

Accumulating evidence has suggested that prenatal exposure to methadone causes multiple adverse effects on human brain development. Methadone not only suppresses fetal neurobehavior and alters neural maturation, but also leads to long-term neurological impairment. Due to logistical and ethical issues of accessing human fetal tissue, the effect of methadone on brain development and its underlying mechanisms have not been investigated adequately and are therefore not fully understood. Here, we use human cortical organoids which resemble fetal brain development to examine the effect of methadone on neuronal function and maturation during early development. During development, cortical organoids that are exposed to clinically relevant concentrations of methadone exhibited suppressed maturation of neuronal function. For example, organoids developed from 12th week till 24th week have an about 7-fold increase in AP firing frequency, but only half and a third of this increase was found in organoids exposed to 1 and 10 μM methadone, respectively. We further demonstrated substantial increases in *I*_*Na*_ (4.5-fold) and *I*_*KD*_ (10.8-fold), and continued shifts of Na^+^ channel activation and inactivation during normal organoid development. Methadone-induced suppression of neuronal function was attributed to the attenuated increase in the densities of *I*_*Na*_ and *I*_*KD*_ and the reduced shift of Na^+^ channel gating properties. Since normal neuronal electrophysiology and ion channel function are critical for regulating brain development, we believe that the effect of prolonged methadone exposure contributes to the delayed maturation, development fetal brain and potentially for longer term neurologic deficits.

## Introduction

Evidence has shown that methadone-exposed fetuses displayed neurobehavioral suppression such as declined motor activity and movement duration, as well as decreased coupling between fetal movement and fetal heart rate in utero (Wouldes et al., 2004; Jansson et al., 2005, 2011). Studies using diffusion MRI found that methadone-exposed newborns exhibited altered neural maturation of connective tracts (Walhovd et al., 2012) and altered microstructure in major white matter tracts (Monnelly et al., 2018). In addition, various investigations showed that children with prenatal methadone exposure have abnormal intellectual capacities and neurobehavioral changes such as lower attention span, higher fear, aggression and anxiety, indicating the existence of long-term neurological impairment caused by in utero methadone exposure (Strauss et al., 1976; Rosen and Johnson, 1982; Bauman and Levine, 1986; Davis and Templer, 1988; Bunikowski et al., 1998; Hunt et al., 2008). Some of these studies also found that children exposed to methadone had impaired cognitive development and language ability (Rosen and Johnson, 1982; Davis and Templer, 1988; Hunt et al., 2008). Therefore, methadone exposure in utero can cause a variety of adverse effects on early brain development.

Human fetal tissues are generally not accessible for direct investigation due to logistical and ethical issues. Previous work mainly relied on animal models to examine the effects of prenatal methadone exposure on brain development. Studies in rats showed that methadone could reduce neurotransmitters and delay neural maturation and synaptogenesis (Seidler et al., 1982; Guo et al., 1990). Another study showed that methadone disrupt brain maturation at early stages of myelination in developing rat brain (Vestal-Laborde et al., 2014). However, the development of human brain has fundamental differences from rodent brain regarding the time course of development, cortical expansion and functional complexity (Semple et al., 2013; Zhao and Bhattacharyya, 2018). Such major differences raise the question whether the findings in rodents can be translated into humans. Therefore, an alternative model that resembles human fetal brain is needed for investigating the impact of opioid exposure in utero on early neurodevelopment. In the past few years, multiple laboratories around the world have developed a three-dimensional (3D) model, named human brain organoids, that are derived from induced pluripotent stem cells (iPSCs) (Watanabe et al., 2007; Eiraku et al., 2011; Lancaster et al., 2013; Pasca et al., 2015; Quadrato et al., 2017; Trujillo and Muotri, 2018; Arlotta and Pasca, 2019; Pasca, 2019; Trujillo et al., 2019; Kim et al., 2020). It has been shown that these brain organoids recapitulate the critical aspects of neuronal function and development of the human fetus at cellular and molecular levels (Lancaster et al., 2013; Pasca et al., 2015; Birey et al., 2017; Quadrato et al., 2017; Trujillo et al., 2019). In addition, recent work from our laboratories and from others have shown the possibility of generating homogenous and reliable brain organoids (Schukking et al., 2018; Velasco et al., 2019; Yoon et al., 2019). Thus, human brain organoids provide a great opportunity to investigate the neurodevelopmental effects of prenatal opioid exposure.

We have previously used the guided method to generate human functional cortical organoids that recapitulate neural network activity and oscillations during early brain development (Trujillo et al., 2019). These cortical organoids can be maintained and developed for prolonged periods in vitro and display complex electrophysiological activity that mimics early human neurodevelopment. In this work, we leveraged our work and took advantage of this opportunity to investigate the effects of methadone, an opioid that has been commonly prescribed or abused during pregnancy, on neural function and maturation. We employed whole-cell patch-clamp techniques to determine whether neuronal electrophysiological function in cortical organoids are affected by long-term methadone exposure at clinically relevant low micromolar concentrations (Wolff et al., 1991).

## Materials and Methods

### Human Cortical Organoids Generation and Culture

Cortical organoids were generated from iPSC lines derived from three control subjects, two males and one female individuals, characterized in previous studies (Nageshappa et al., 2016; Trujillo et al., 2019; Yao et al., 2020). The iPSCs used in this study had been passaged for 20–30 times and have been routinely checked by karyotype and CNV arrays to check on genomic alterations in culture. Human cortical organoids were generated and maintained using the same protocol as previously described (Nageshappa et al., 2016; Trujillo et al., 2019; Yao et al., 2020). Briefly, the feeder-free iPSCs were cultured with mTeSR1 media for 7–8 days. Then, accutase (Life Technologies) in PBS (1:1) were used to dissociate the iPSCs colonies at 37°C for 15–20 min. The cells were further centrifuged for 3 min at 500 rpm and resuspended in mTeSR1 media supplemented with 10 μM SB and 1 μM Dorsomorphin. To have the 3D free-floating spheres, the iPSCs were transferred to a 6-well plate (∼4 × 10^6^ cells per well) and kept in suspension by adding 5 μM ROCK inhibitor for 24 h. Next, the spheres were maintained in media1 [Neurobasal (Thermo Fisher Scientific, Waltham, MA, United States) supplemented with 1× Glutamax, 1× Gem21, 1× N2, 1× NEAA (Life Technologies, Carlsbad, CA, United States), 1× PS, 10 μM SB and 1 μM Dorso] for 7 days. Media1 was then substituted by Media2 (Neurobasal with 1× Glutamax, 1× Gem21, 1× NEAA, and 1× PS), plus 20 ng/mL bFGF for 7 days. Furthermore, the spheres were maintained in Media3 [Media2 supplemented with 10 μg/mL of BDNF, 10 μg/mL of GDNF, 10 μg/mL of NT-3 (PeproTech, Rocky Hill, NJ, United States), 200 μM L-ascorbic acid and 1 mM dibutyryl-cAMP (Sigma-Aldrich, St. Louis, MO, United States)] for 7 days. Afterward, the cortical organoids were formed and maintained as long as needed in Media2.

### Solutions and Drugs

The extracellular solution for patch-clamp experiments contained (in mM): 130 NaCl, 3 KCl, 1 CaCl_2_, 1 MgCl_2_, 10 HEPES, and 10 glucose; pH 7.4 with 1 M NaOH (∼4 mM Na^+^ added). The internal solution for patch electrodes contained (in mM): 138 K-gluconate, 4 KCl, 10 Na_2_-phosphocreatine, 0.2 CaCl_2_, 10 HEPES (Na^+^ salt), 1 EGTA, 4 Mg-ATP, 0.3 Na-GTP; pH 7.4 with 1 M KOH (∼3 mM K^+^ added). In some whole-cell recordings, the internal solution also includes 1 mg/ml Lucifer yellow dye. The osmolarity of all solutions was adjusted to 290 mOsm. Methadone (Sigma-Aldrich, St. Louis, MO, United States) was dissolved in water to make 1 mg/mL stock solutions. Methadone was diluted in Media2 to working concentrations (1 and 10 μM). The cell culture media (Media2 + methadone) was filtered with Tube-Top Vacuum Filter (Corning, Tewksbury, MA, United States) to ensure sterility.

### Whole-Cell Patch-Clamp Recordings and Analysis

To perform patch-clamp recordings, 1–2 cortical organoids at the age of 10 weeks were plated on 35 mm dishes and were continuously attached in the maintaining media (Media2) for up to the 24th week, with media changes 2–3 times per week. To minimize the variability among individual cells, whole-cell patch-clamp recordings were performed from cells (Figure 2A) within a fixed range (<500 μm) from the edge of cortical organoids in the dish. In general, 1–3 cells were patched per organoid in each condition, i.e., dose and timepoint of methadone treatment. Electrodes for electrophysiological recording were pulled on a Flaming/Brown micropipette puller (Model P-87, Sutter Instrument, Novato, CA, United States) from filamented borosilicate capillary glass (1.2 mm OD, 0.69 mm ID, World Precision Instruments, Sarasota, FL, United States). The electrode resistances were 6–10 MΩ for whole-cell recording. Patch clamp experiments were performed with an Axon CV-4 headstage and Axopatch 200 A amplifier (Molecular Devices, San Jose, CA, United States) at room temperature. The same electrophysiological protocol was used to all cells at the age of 12-, 16-, 20-, and 24-week-old organoids. After breaking the membrane seal, we first measured *C*_*m*_ and *R*_*in*_ in voltage-clamp mode. Second, we switched to current-clamp configuration to record the *V*_*rest*_ and action potential firings. Small currents were injected to maintain the membrane potential around −70 mV for evoked AP recordings. Then, we switched back to voltage-clamp mode to record the currents of voltage-dependent Na^+^ and K^+^ channels. Recordings were low-pass filtered at 1 kHz, and digitized at 10 kHz using a DigiData 1322A (Molecular Devices, San Jose, CA, United States). The voltages used were corrected by the liquid junction potentials (10 mV). Electrophysiology data were analyzed offline using pCL AMP 10 software (Molecular Devices, San Jose, CA, United States).

Peak Na^+^ currents were calculated with pulses between −100 and +20 mV from a holding potential of −100 mV. The peak Na^+^ currents were plotted against voltage to have the current–voltage relationships. The Na^+^ channel conductance was assessed from the peak *I*_*Na*_ amplitude using the formula: *G* = *I*_*Na*_/(V – *E*_*rev*_), where G is channel conductance, *I*_*Na*_ is peak current size measured at test potential V, *E*_*rev*_ is the estimated Na^+^ reversal potential, and then fit with the Boltzmann function to have the maximal conductance. The voltage dependence of Na^+^ channel activation curves were generated by normalizing to the maximal conductance. To measure the voltage dependence of Na^+^ channel availability (i.e., inactivation), cells were recorded following a 200-ms pre-pulse to various potentials from −140 to −20 mV and normalizing to the peak current measured with a pulse to 0 mV. The normalized G-V curves for channel activation or inactivation were fit with the Boltzmann function: *G* = 1/[1 + exp(V − V_1/2_)/k] to determine the V_1/2_ and slope factor (k).

### Immunocytochemistry

Organoids were fixed with 4% paraformaldehyde for 30 min on ice and washed three times with PBS before incubated in 30% Sucrose solution at 4°C overnight. Cryoprotected organoids were then embedded in OCT followed by flash freezing. The frozen organoids were sectioned into 12 μm-thick slices for immunofluorescence staining, which was carried out after blocking samples in a solution with 0.1% Triton (Sigma-Aldrich) and 3% BSA (Sigma-Aldrich) for 30 min at room temperature. The primary antibodies were diluted in a solution with 0.1% Triton and 3% BSA, and the sections were incubated with the following antibodies: anti-SOX2 (rabbit, Cell Signaling Technology 2748, 1:500), anti-CTIP2 (rat, Abcam, ab18465, 1:500), and anti-MAP (chicken, Abcam, ab5392, 1:2000). The secondary antibodies used were Alexa 488, Alexa 555 and Alexa 647 conjugated to specific IgG types (Invitrogen Molecular Probes, 1:1000) for 2 h at room temperature. Nuclei were visualized using 4 ’,6-diamidino2-phenylindole, dihydrochloride (DAPI) counterstain (Sigma-Aldrich, St. Louis, MO, United States). Samples were covered with thin coverglass followed by image capture using an Olympus FV1000 confocal microscopy (Olympus Inc., Center Valley, PA, United States).

To confirm cell identity in cortical organoids, we combined whole-cell patch clamp recordings with immunocytochemistry. Cells were double labeled with Lucifer yellow and a fluorescence-labeled antibody such as VGLUT1 or GFAP. Presumed glutamatergic neuron or astrocyte were filled with Lucifer yellow during whole-cell recordings. The cortical organoid was then fixed in 4% paraformaldehyde (pH 7.4) for 20 min, washed, and processed for immunoreactivity at room temperature (25). The primary antibodies were polyclonal rabbit anti-VGLUT1 (Synaptic Systems, Goettingen, Germany) and monoclonal mouse anti-GFAP (Thermo Fisher Scientific, Waltham, MA, United States). The secondary antibodies were goat anti-rabbit and goat anti-mouse IgG highly cross-adsorbed antibodies (Thermo Fisher Scientific, Waltham, MA, United States). The fixed and stained organoids were mounted in ProLong Gold Antifade Reagent with DAPI (Life Technologies, Carlsbad, CA, United States) and viewed using a Zeiss inverted Axiovert 200 M microscope.

### Statistical Analysis

Where appropriate, data were analyzed with Student’s *t*−test, one-way ANOVA, or two-way ANOVA. Significance was defined as *P* < 0.05 (^∗^ or ^#^), *P* < 0.01 (^∗∗^ or ^##^), *P* < 0.001 (^∗∗∗^ or ^###^), *P* < 0.0001 (^****^ or ^####^). All error bars in figures are SEM.

## Results

**FIGURE 1:**
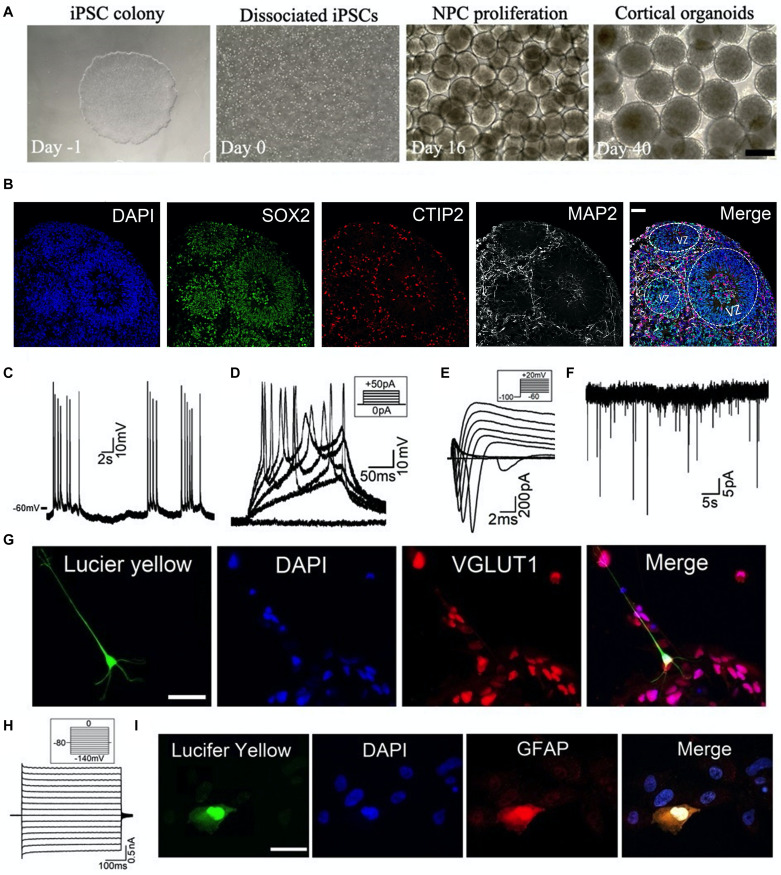
Generation and characterization of human cortical organoids. **(A)** Schematic of cortical organoid generation from dissociation of iPSCs (day -1, 0) to neural induction (day 16) and then to neural proliferation and maturation (day 40). Scale bar, 500 μm. **(B)** Typical immunostaining images on cryosections of 2-month-old cortical organoid showing the neural differentiation profile. VZ, ventricular zoon. Scale bar, 50 μm. **(C–I)** Combining patch-clamp recordings with immunocytochemical staining to define cell types of cortical organoids. **(C–F)** Whole-cell patch-clamp recordings of **(C)** spontaneous Action Potential (AP) firing, **(D)** evoked AP firing, **(E)** voltage-gated Na^+^ currents (*I*_*Na*_) and K^+^ currents (*I*_*K*_), and **(F)** spontaneous excitatory postsynaptic currents (sEPSCs) voltage-clamped at –60 mV, from a representative neuron located at the edge of a 3-month-old organoid in the dish. Recording protocols were shown in the inset. **(G)** The same neuron was filled with Lucifer yellow (1 mg/ml) during recording (left), and then this organoid was processed for immunocytochemistry with glutamatergic neuron marker VGLUT1. Merged labeling (right) confirmed that the recorded cell was a glutamatergic neuron. Scale bar, 50 μm. **(H)** Patch-clamp recording of weakly inward rectifying K^+^ currents (*I*_*Kir*_) in a glial cell located at the edge of a 3-month-old organoid. **(I)** The cell was filled with Lucifer yellow (1 mg/ml) during recording (left), and then this organoid was processed for immunocytochemistry with astrocyte marker GFAP. Merged labeling (right) confirmed that the recorded glial cell was an astrocyte. Scale bar, 50 μm.

**FIGURE 2:**
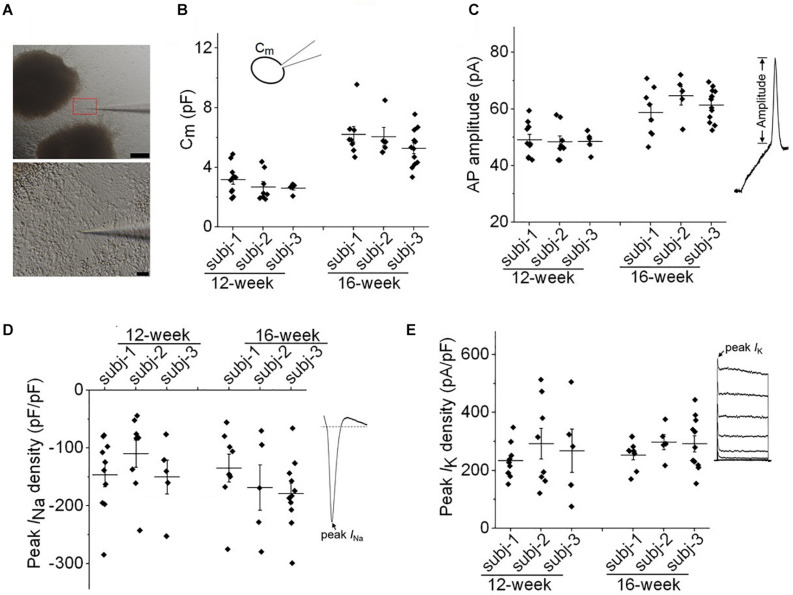
Evaluation of variability in neuronal membrane properties. **(A)** Representative images of whole-cell patch clamp recording of a neuron at the edge of cortical organoids. Scale bar, 200 μm (upper panel) and 10 μm (lower panel). **(B,C)** Plots of cell capacitance (*C*_*m*_), and AP amplitude (evoked by 50 pA) of individual neurons from three healthy subjects (subj-1, subj-2, and subj-3) for 12-week and 16-week old organoids. For both panels, one-way ANOVA, *p* > 0.99 for comparisons among three subjects at 12-week, or 16-week. *n* = 5–12 neurons. **(D)** Plot of peak *I*_*Na*_ density of individual neurons from three healthy subjects at the age of 12-week and 16-week. One-way ANOVA, *p* = 0.36. *n* = 5–11 neurons. **(E)** Plot of peak *I*_*K*_ density of individual neurons. One-way ANOVA, *p* = 0.73. *n* = 5–11 neurons. Recording protocols for *I*_*Na*_ and *I*_*K*_ were shown in Figures 5, 6. Graphs display mean and SEM.

Human cortical organoids were generated from iPSC lines as described in our recent work (Nageshappa et al., 2016; Trujillo et al., 2019; Yao et al., 2020). As shown in Figure 1A, the iPSCs were dissociated into individual cells which then form 3D aggregates for further neural differentiation and maturation on the shaker. We used specific markers to determine the differentiation profile on cryosections of cortical organoids. As shown in Figure 1B, we observed an abundance of neural progenitor cells (SOX2+) in the ventricular zoon-like (VZ) region in the 2-month-old cortical organoid. These SOX2+ cells were surrounded by deep layer neurons (CTIP2+) and more matured neurons (MAP2+) in the sub-ventricular zone and cortical plate-like regions, which resembles the human brain corticogenesis *in vivo*. Further, we have combined whole-cell patch-clamp techniques and immunocytochemistry to define cell types and their electrophysiological properties. To perform patch-clamp recordings, one or two cortical organoids were plated in each dish to allow cell growth and maturation (Figure 2A). The internal solution in the patch-clamp electrode was filled with Lucifer yellow dye to label the cells we recorded from, and this will serve to identify cells for further immunocytochemistry. As shown in Figures 1C–G, a representative neuron, located at a ∼100 μm distance from the edge of a 3-month-old organoid, exhibited (1) spontaneous and evoked action potential (AP) firing, (2) voltage-dependent Na^+^ and K^+^ currents (*I*_*Na*_, *I*_*K*_), and (3) spontaneous excitatory postsynaptic currents (sEPSCs). Further immunocytochemical staining of the same organoid verified that this neuron is a glutamatergic neuron (Figure 1G). Using the same approach, we recorded from a glial cell showing weakly inward rectifying K^+^ current (*I*_*Kir*_) and further immunocytochemical staining confirmed that the cell we recorded from is a GFAP-immunoreactive astrocyte (Figures 1H,I). These results demonstrate that specific types of cells at the edge of cortical organoids can be identified by combining patch-clamp recordings and immunocytochemistry. Our findings also confirm that this glutamatergic neuron and this astrocyte are functionally active in human cortical organoids.

We further evaluated the variabilities of electrophysiological properties in cortical organoids among three healthy subjects. As shown in Figure 2A, we chose to patch cells within a consistent distance (<200 μm) from the edge of cortical organoids in all patch-clamp recordings. Both passive and active membrane properties were used to assess variability. In Figures 2B,C, one-way ANOVA analysis demonstrated no significant difference among three subjects regarding cell capacitance (*C*_*m*_) or AP amplitude of individual neurons at the age of 12-week or 16-week. For the same subject, the *C*_*m*_ or AP amplitude in neurons of 16-week-old organoids were significantly higher than that of 12-week (*p* < 0.01, one-way ANOVA followed by Bonferroni *post hoc* test) indicating a developmental growth in cell size and AP amplitude. In Figures 2D,E, one-way ANOVA analysis showed no significant change in *I*_*Na*_ or *I*_*K*_ densities among all groups, indicating a consistency of neuronal properties among three iPSC lines.

**FIGURE 3:**
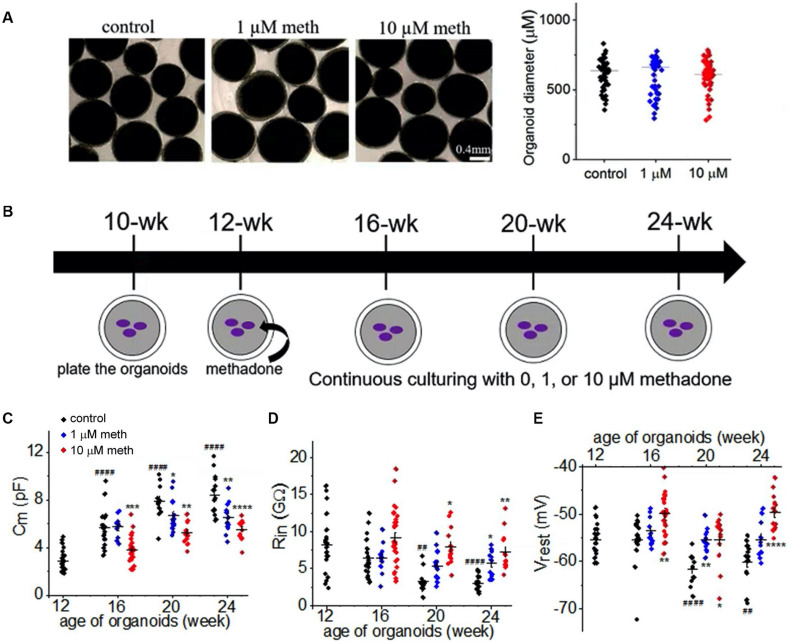
Methadone exposure suppresses the development of the passive neuronal properties. **(A)** Bright field images of organoids in suspension in control and methadone treatment groups from 12-week till 16-week. 4 weeks treatment of methadone at 1 and 10 μM do not significantly alter organoid diameters. **(B)** Schematic of the time course for the culture and methadone treatment of cortical organoids in the dish. Whole-cell patch-clamp recordings were performed for every 4-week from 12-week, till 24-week. **(C–E)** Scatter-plots of the *C*_*m*_, input resistance (*R*_*in*_) and resting membrane potential (*V*_*rest*_) recording of neurons from cortical organoids during the early development (12- to 24-week) in the absence and presence of 1 or 10 μM methadone. In each scatter plot, Black, Blue, and Red dots denote untreated, 1 and 10 μM methadone group, respectively. Graphs display mean and SEM. *n* = 10–29 neurons (7–17 organoids). Two-way ANOVA followed by Tukey’s multiple comparisons test: **(C)** *F*_(6, 162)_ = 6.26, *p* < 0.0001; **(D)** *F*_(6, 162)_ = 1.92, *p* = 0.08; **(E)** *F*_(6, 162)_ = 6.11, *p* < 0.0001. Control groups at 16-week, 20-week, 24-week were compared to 12-week group, ^##^*p* < 0.01, ^####^*p* < 0.0001. Each methadone-treated groups were compared to the control group at the same age, *p < 0.05, **p < 0.01, ***p < 0.001, ****p < 0.0001.

Consistent with a previous study (Stoetzer et al., 2015), acute application of methadone at clinically relevant concentrations (low micromolar) caused no significant changes in the function of neuronal Na^+^ channel in cortical organoids (data not shown). To investigate the *chronic* effect of methadone exposure on neuronal properties during early development, we took advantage of the human organoid model which can be maintained for prolonged period of time (a few months) (Trujillo et al., 2019). As demonstrated in Figure 3B, human cortical organoids were plated at the age of 10 weeks in the dish and maintained till 24 weeks. These cortical organoids were treated with methadone (1 or 10 μM) from 12 weeks till 24 weeks. At these doses and during that period, organoids in suspension did not have any apparent change in size or integrity (Figure 3A). We performed parallel patch-clamp recordings of cortical organoids for control and methadone-treated groups. Neurons were distinguished from other types of cells based on their functionalities: AP firing and voltage-dependent Na^+^ current. During the developmental period, we found a progressive increase of membrane surface area (*C*_*m*_) and a gradual reduction of input resistance (*R*_*in*_) in neurons in the control group (Figures 3B,C and Table 1). However, methadone exposure at 1 and 10 μM concentrations caused a significant attenuation in the growing *C*_*m*_ and the reduction in *R*_*in*_ compared with those in the control group. In addition, methadone exposure from 12- to 24-weeks induced a significant depolarization of resting membrane potential (*V*_*rest*_) in Figure 3D and Table 1. These data suggest that methadone exposure at low micromolar concentrations suppresses the progressive alterations in the subthreshold membrane properties during early development.

**TABLE 1:** Descriptive statistics of the 15 electrophysiological parameters in neurons recorded at the edge of cortical organoids.

Age, treatment	*C*_*m*_ (pF)		*R*_*m*_ (GΩ )		*V*_*rest*_ (mV)		AP frequency (Hz)		AP amplitude (mV)		AP ½ width (ms)		AP threshold (mV)		AP rise slope (mV/ms)		*N*
									
	Mean	SD	Mean	SD	Mean	SD	Mean	SD	Mean	SD	Mean	SD	Mean	SD	Mean	SD	
12-wk, control	2.89	0.91	8.15	3.77	–55.3	3.1	1.73	1.16	48.7	5.5	7.59	3.30	–27.6	2.7	31.8	9.2	21–23
16-wk, control	5.62	1.50	6.40	2.53	–55.3	4.7	4.05	1.98	60.8	7.5	3.17	0.82	–25.9	3.3	56.1	12.5	15–20
16-wk, 1 μM meth	5.73	0.94	6.41	2.13	–53.6	3.0	4.25	1.67	60.6	6.5	3.26	0.66	–25.2	1.7	56.0	9.9	8–10
16-wk, 10 μM meth	3.82	1.04	9.19	3.70	–49.8	4.8	1.79	0.98	50.0	6.2	4.52	1.58	–22.9	2.4	43.0	9.5	19–29
20-wk, control	7.85	1.42	3.23	1.52	–61.7	3.2	13.00	2.48	62.9	6.1	2.62	0.75	–27.7	3.8	63.5	12.4	9–12
20-wk, 1 μM meth	6.7	1.37	5.33	2.04	–55.4	2.3	6.85	3.7	59.6	8.4	3.03	1.30	–24.9	2.4	60.8	9.7	11–14
20-wk, 10 μM meth	5.24	0.96	7.89	2.45	–55.3	5.1	2.54	1.13	51.1	5.3	3.52	0.78	–24.3	3.3	47.1	10.4	13–14
24-wk, control	8.39	1.65	2.98	0.99	–60.1	4.0	11.72	4.08	68.1	8.0	2.56	0.90	–33.1	2.6	74.7	20.6	14–15
24-wk, 1 μM meth	6.49	1.28	5.70	1.68	–55.4	3.8	6.55	3.53	66.2	7.2	3.20	1.13	–28.9	2.9	66.9	13.7	11–12
24-wk, 10 μM meth	5.51	0.84	7.25	2.59	–49.6	3.9	4.73	2.41	62.2	10.3	3.29	1.07	–30.7	2.5	54.2	12.5	11–13

**Age, treatment**	**AP decay slope (mV/ms)**			**AHP amplitude (mV)**			**peak I*Na* (pA)**		**V0:5-activ (mV)**		**V0:5-inactiv (mV)**		**peak I*KD* (pA)**		**peak I*KA* (pA)**		***N***
								
	**Mean**		**SD**	**Mean**		**SD**	**Mean**	**SD**	**Mean**	**SD**	**Mean**	**SD**	**Mean**	**SD**	**Mean**	**SD**	

12-wk, control	–16.1		8.6	5.2		4.5	–394.7	233.1	–34.0	4.3	–50.0	3.7	201.5	140.6	528.2	253.7	21–23
16-wk, control	–41.7		7.5	9.8		2.6	–846.9	329.6	–37.3	2.8	–38.7	3.7	966.1	211.6	644.2	160.7	15–20
16-wk, 1 μM meth	–44.4		8.8	10.9		3.4	–815.3	468.3	–35.1	1.7	–40.5	2.4	704.1	768.7	466.7	557.2	8–10
16-wk, 10 μM meth	–32.1		6.9	4.7		3.3	–463.7	236.3	–31.3	3.5	–40.8	4.3	594.2	251.1	526.1	210.9	19–29
20-wk, control	–48.8		9.0	14.3		4.3	−1, 513.0	593.3	–38.6	5.4	–36.6	4.8	1, 738.1	834.8	982.1	454.5	9–12
20-wk, 1 μM meth	–49.5		8.6	13.8		4.2	−1, 095.3	377.4	–34.4	4.9	–37.7	6.0	1, 162.7	485.9	1, 012.5	495.6	11–14
20-wk, 10 μM meth	–36.1		6.1	7.8		3.5	–615.1	270.1	–33.8	5.7	–42.4	3.1	651.4	183.2	661.9	391.3	13–14
24-wk, control	–54.7		15.1	16.4		4.1	−1, 766.9	476.7	–45.6	4.9	–38.9	3.4	2, 164.8	733.2	930.2	263.3	14–15
24-wk, 1 μM meth	–46.1		8.1	12.4		3.8	−1, 061.4	423.8	–40.0	3.8	–41.6	4.1	1, 208.2	494.3	774.5	283.6	11–12
24-wk, 10 μM meth	–38.9		5.3	12.6		3.9	–850.3	241.5	–39.0	5.3	–42.5	5.7	922.3	361.2	677.6	337.2	11–13

**C*_*m*_, membrane capacitance; *R*_*in*_, input resistance, *V*_*rest*_, resting membrane potential; AP, action potential; AHP, afterhyperpolarization; V_0.5–activ_, half-maximal activation voltage of Na^+^ channels; V_0.5–inactiv_, half-maximal inactivation voltage of Na^+^ channels.*

**FIGURE 4:**
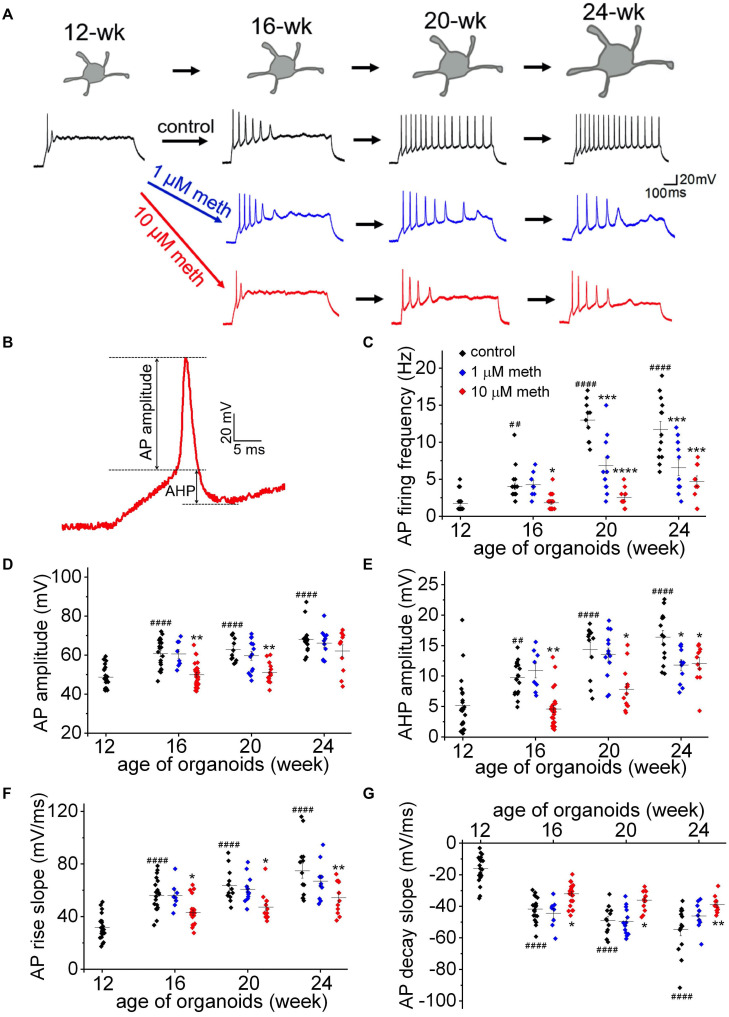
Methadone exposure suppresses the increase in membrane excitability during early neurodevelopment. **(A)** Representative traces of AP firing in neurons from human cortical organoids at indicated ages (12- to 24-week) in the absence and presence of methadone treatments. APs were evoked from –70 mV to 1,000 ms-long injections of 50 pA current. The exposure of 1 and 10 μM methadone hinders the growth of AP firing during the indicated period of cortical organoids. **(B)** Representative trace of the 1st AP for the measurement of AP and AHP amplitudes. **(C)** Summary of the AP firing frequency in response to 1,000 ms current step (50 pA) from –70 mV in neurons at the edge of organoids at indicated ages. **(D–G)** Summary of the AP amplitude, AP maximal rise slope, AP maximal decay slope, and AHP amplitude evoked by the same current injection (50 pA). In each scatter plot, Black, Blue, and Red dots denote untreated, 1 and 10 μM methadone treated group, respectively. Graphs display mean and SEM. *n* = 8–25 neurons (6–15 organoids). Two-way ANOVA followed by Tukey’s multiple comparisons test: (C) *F*_(6, 162)_ = 15.47, *p* < 0.0001; **(D)** *F*_(6, 162)_ = 3.11, *p* < 0.01; **(E)** *F*_(6, 162)_ = 3.67, *p* < 0.01; **(F)** *F*_(6, 162)_ = 2.74, *p* < 0.05; **(G)** *F*_(6, 162)_ = 3.62, *p* < 0.01. Control groups at 16-week, 20-week, 24-week were compared to 12-week group, ^##^*p* < 0.01, ^####^*p* < 0.0001. Each methadone-treated groups were compared to the control group at the same age, *p < 0.05, **p < 0.01, ***p < 0.001, and ****p < 0.0001.

To directly investigate the effect of methadone exposure on membrane excitability during early development, we measured the AP firing frequency (evoked by a 50 pA current injection for 1,000 ms), as well as six single AP properties including AP amplitude, AP half-width, AP threshold, AP maximal rise slope, AP maximal decay slope, and afterhyperpolarization (AHP) amplitude (Figure 4 and Table 1). As shown in Figure 4A, we found progressive changes in AP firing frequency and single AP properties from 12-week to 24-week old organoids in the control group, indicating a “maturing” process of neuronal membrane excitability. However, the long term methadone exposure at 1 and 10 μM concentrations caused a significant attenuation of the progressive increase in the AP firing frequency, the AP amplitude, the maximal AP rise/decay slope, and the AHP amplitude (Figures 4B–G and Table 1), suggesting that methadone suppresses the intrinsic neuronal excitability in a concentration- and time-dependent way. The results in Figures 3, 4 indicate that chronic methadone exposure at clinically relevant concentrations lead to a longitudinal impairment of membrane properties and neuronal excitability during early brain development.

**FIGURE 5:**
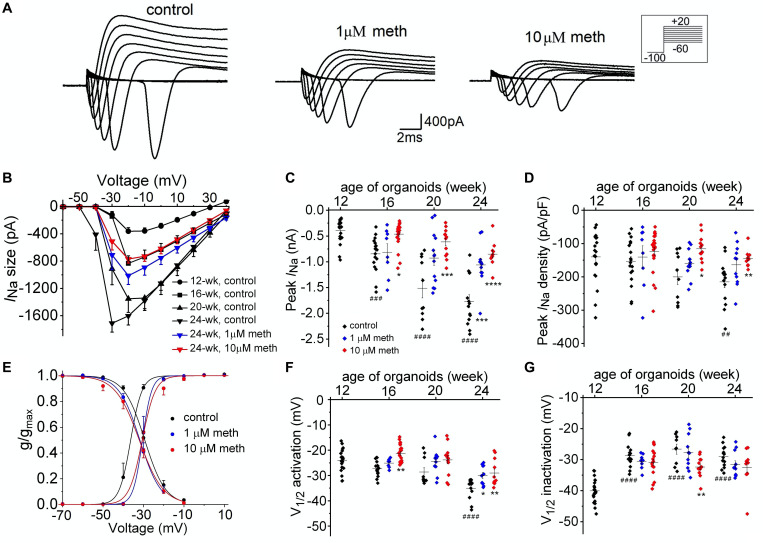
Methadone exposure attenuates the developmental change of Na^+^ current properties. **(A)** Na^+^ current traces recorded from representative neuronsfrom 16-week-old organoids in control and 4-week treatments of 1 μM and 10 μM methadone. **(B)** Plot of average Na^+^ current size as a function of voltage determined from neurons at the edge of cortical organoids at indicated ages and methadone treatments. Chronic methadone exposure significantly attenuated the developmental increase of *I*_*Na*_ size. **(C,D)** Scatter plot showing the peak *I*_*Na*_ size **(C)** and *I*_*Na*_ density **(D)** of neurons from human cortical organoids with indicated ages and methadone treatments. **(E)** Voltage dependence of activation and inactivation curves calculated for Na^+^ currents recorded from 24-week-old neurons of cortical organoids in control groups and methadone treated groups. The solid lines represent Boltzmann fits to the average data. **(F,G)** Scatter plot showing voltages at which half of the Na^+^ channels were activated **(F)** or inactivated **(G)** in each group. Graph of scatter plots display mean and SEM. *n* = 8–24 neurons (6–15 organoids). Two-way ANOVA followed by Tukey’s multiple comparisons test: **(C)** *F*_(6, 162)_ = 7.39, *p* < 0.0001; **(D)** *F*_(6, 162)_ = 1.38, *p* = 0.22; **(F)** *F*_(6, 162)_ = 2.23, *p* < 0.05; **(G)** *F*_(6, 162)_ = 1.93, *p* = 0.06. Control groups at 16-week, 20-week, 24-week were compared to 12-week group, ^##^*p* < 0.01, ^###^*p* < 0.001, and ^####^*p* < 0.0001. Each methadone-treated groups were compared to the control group at the same age, *p < 0.05,**p < 0.01, ***p < 0.001, ****p < 0.0001.

Since AP shape and firing pattern in neurons are defined by voltage-dependent Na^+^ and K^+^ currents, we further investigated the functional changes of these ion currents in the presence and absence of methadone. We used voltage clamp techniques to measure the ionic currents (*I*_*Na*_ and *I*_*K*_) in neurons from 12-week to 24-week of organoid age. As shown in Figures 5A,B, peak Na^+^ currents were measured using voltage steps to have the current-voltage relationships for each group. We observed a remarkable increase in neuronal *I*_*Na*_ size during this developmental period (Figures 5B,C) in the control group. Since the increase in *I*_*Na*_ size is larger than that in the *C*_*m*_ (Figures 3B, 5C), the Na^+^ current density also gradually increased during the same time course (Figure 5D). In contrast, cortical organoids exposed to 1 and 10 μM methadone exhibited significant reduction of the increase in neuronal *I*_*Na*_ size/density during this time period. For example, the peak *I*_*Na*_ density was increased from −117.5 ± 12.4 pA/pF in 12-week organoids to −205.7 ± 17.6 pA/pF in 24-week organoids in control groups, whereas peak *I*_*Na*_ density in 10 μM methadone treated group only reached −133.5 ± 7.0 at 24-week (Figure 5D). Thus, the methadone-induced suppression of intrinsic neuronal excitability seen in Figure 4 was contributed, at least partially, by the significant reduction of *I*_*Na*_ size and density.

Next, we explored the effect of methadone on biophysical properties of neuronal Na^+^ channels from 12- to 24-week-old organoids. During such developmental period in control groups, as shown in Figures 5E–G, we found an “earlier” opening of Na^+^ channels due to a negative shift of voltage dependence of activation and a large increase of Na^+^ channel availability due to the positive shift of voltage dependence of inactivation. In the methadone-treated group, however, the alterations of voltage dependence of activation and inactivation were significantly blunted. For example, from week 12 to week 24, the *V*_1/2_ value for activation was reduced from −32.2 ± 1.2 mV to −45.6 ± 1.4 mV in the control group (Student’s *t*-test: *n* = 14–21, *p* < 0.0001) while the *V*_1/2_ value was decreased to −39.0 ± 1.6 mV in 10 μM methadone group (Student’s *t*-test: *n* = 11, *p* < 0.01, compared to 24-week control) as seen in Figures 5E,F and Table 1. During the same time period, channel availability at −50 mV was increased from 47.5 ± 5.8% to 90.5 ± 1.6% in the control group (Student’s *t*-test: *n* = 14–21, *p* < 0.0001), but to only 80.0 ± 5.7% in the 10 μM methadone group (Student’s *t*-test: *n* = 11, *p* < 0.05, compared to 24-week control). Thus, the altered gating properties (both activation and inactivation) of Na^+^ channels have a significant contribution to the maturation of AP firings during this period. The attenuation of the increase in *I*_*Na*_ size/density and the shifts of gating properties of Na^+^ channels was responsible for the methadone-induced suppression of intrinsic neuronal excitability in human cortical organoids.

**FIGURE 6:**
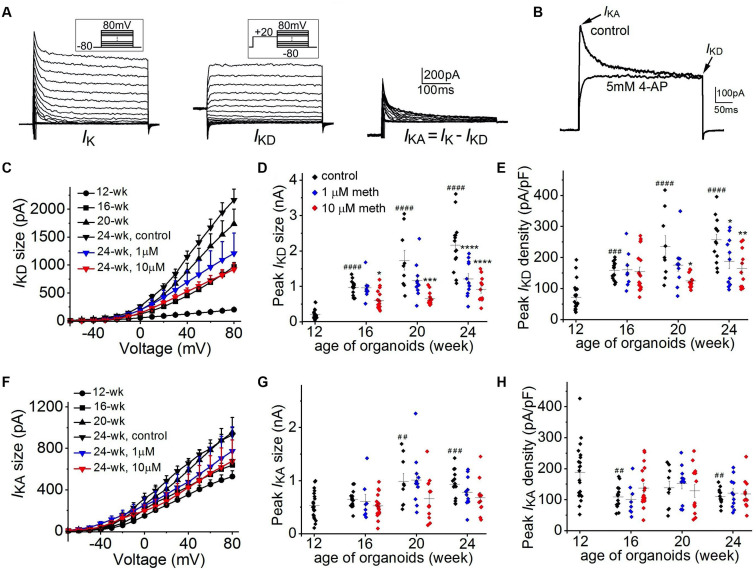
Methadone attenuates the developmental increase of non-inactivating K^+^ currents. **(A)** K^+^ current traces recorded from a representative neuron at the edge of 16-week-old organoids without methadone treatment. Left panels, the overall potassium current (*I*_*K*_) recorded at voltages from –80 to +80 mV. Middle panels, non-inactivating delayed-rectifier K+ current (*I*_*KD*_) preceded by a prepulse to +20 mV. Right panels, transient A-type current (*I*_*KA*_) obtained by subtracting the *I*_*KD*_. **(B)** Neuronal K^+^ current traces (depolarized at +20 mV from holding potential of –80 mV) recorded from another neuron before and after the application of 5 mM 4-AP. **(C)** Plot of *I*_*KD*_ size as a function of voltage determined from neurons of cortical organoids at indicated ages and methadone treatments. **(D,E)** Scatter plot showing the peak *I*_*KD*_ size **(D)** and density **(E)** of neurons from human cortical organoids with indicated ages and methadone treatments. **(F)** Plot of *I*_*KA*_ size as a function of voltage determined from neurons of cortical organoids at indicated ages and methadone treatments. **(G,H)** Scatter plot showing the peak *I*_*KA*_ size **(G)** and density **(H)** of neurons from human cortical organoids with indicated ages and methadone treatments. Graph of scatter plots display mean and SEM. *n* = 8–19 neurons (6–12 organoids). Two-way ANOVA followed by Tukey’s multiple comparisons test: **(D)** *F*_(6, 162)_ = 10.80, *p* < 0.0001; **(E)** *F*_(6, 162)_ = 3.80, *p* < 0.01; **(G)** *F*_(6, 162)_ = 1.34, *p* = 0.24; **(H)** *F*_(6, 162)_ = 0.45, *p* = 0.84. Control groups at 16-week, 20-week, 24-week were compared to 12-week group, ^##^*p* < 0.01, ^###^*p* < 0.001, ^####^*p* < 0.0001. Each methadone-treated groups were compared to the control group at the same age, *p < 0.05, **p < 0.01, ***p < 0.001, ****p < 0.0001.

Further, we investigated the functional changes in neuronal K^+^ channels from 12-week to 24-week of organoid age. As shown in Figure 6A, neuronal K^+^ currents are composed of two distinct components: a non-inactivating delayed rectifier current (*I*_*KD*_) and an A-type transient, inactivating current (*I*_*KA*_). As shown in Figure 6B, 4-AP (5 mM), a specific blocker for *I*_*KA*_ was applied in another neuron and the transient A-type K^+^ current was fully inhibited, confirming the existence of two components of neuronal K^+^ currents in cortical organoids. Overall, for the 12-week-old organoids, neuronal K^+^ currents are composed of a dominant *I*_*KA*_ (528.2 ± 55.4 pA at +80 mV) while *I*_*KD*_ (201.5 ± 30.7 pA at +80 mV) is relatively small (Figures 6C–F). With the increasing organoid age, we found a continuous growth in *I*_*KA*_ size and a much larger increase in *I*_*KD*_ size in these corticoids. For example, from 12-week to 24-week, there was a >10-fold increase of peak *I*_*KD*_ (2164.8 ± 195.9 pA) but about a 2-fold increase in peak *I*_*KA*_ (930.2 ± 65.3 pA) was observed (Figures 6D–G) in the control group, suggesting a major role of *I*_*KD*_ for AP repolarization during organoid development. Continuous exposure of cortical organoids to methadone in the same period induced a significant reduction of the progressive increase of *I*_*KD*_ size (Figures 6C,D), but no significant changes on *I*_*KA*_ size (Figures 6F,G). Since the reduction of *I*_*KD*_ size is larger than the change in *C*_*m*_, *I*_*KD*_ densities in 1 and 10 μM methadone-treated groups at 20-week and 24-week were significantly lower than those in the control groups (Figures 6D,E). In contrast, *I*_*KA*_ densities were not significantly altered in the methadone-treated groups during the time course (Figure 6H). Our results showed that methadone exposure specifically hindered the growth of the non-inactivating component of neuronal K^+^ currents (*I*_*KD*_) during early neurodevelopment whereas the inactivating component (*I*_*KA*_) was not significantly affected. Since *I*_*KD*_ is the major driving force for AP repolarization during organoid development, the results here indicate that methadone-induced suppression of neuronal excitability is largely attributed to the attenuation of the increase in the non-inactivating component.

**FIGURE 7:**
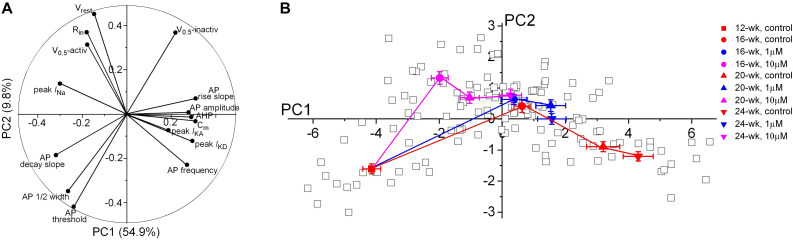
Principal component analysis of electrophysiological properties of neurons during early development. **(A)** Polar plot showing the respective contribution of each of the 15 electrophysiological parameters to the two principal components retained from the PCA (PC1 and PC2). **(B)** Scatter plot representing the factor loadings of the 132 neurons and the averaged factor loadings for each age and each methadone treatment of cortical organoids. Error bars represent the standard error of the mean. This PCA integrates intrinsic electrophysiological properties including passive membrane properties, APs frequency and properties, current sizes and gating properties of Na^+^ and K^+^ channels. Glia or other non-functional cells were not included in the PCA. Neurons that did not have unambiguous analysis of all the chosen 15 electrophysiological properties were also excluded from this PCA analysis.

Since a broad range of intrinsic neuronal properties were changed in a time- and concentration-dependent manner (Figures 3–6), we applied principal component analysis (PCA) as an unbiased statistical analysis to summarize the data variations and to show a precise visualization of the developmental trajectory of the electrophysiological features in developing neurons. Our PCA analysis was based on 15 electrophysiological parameters reflecting the intrinsic properties of 132 patch-clamped neurons from 12- to 24-week organoids (Figure 7 and Table 1). The first principal component (PC1) is the major variance, accounting for 54.9% of the overall variation of our observations as compared to 9.8% in PC2 component. The contribution of each electrophysiological parameter to PC1 and PC2 is demonstrated in Figure 7A. *C*_*m*_, peak *I*_*Na*_, the rise and decay of AP stand out as four of the most influential variables in PC1, indicating a major role of these features during electrophysiological development. In contrast, peak *I*_*KA*_ showed small contributions to both PC1 and PC2. As shown in Figure 7B, the progressive shift of the major variable, PC1 values, in the control group from 12-week to 24-week indicates a developmental trajectory of the electrophysiological features in those neurons. The methadone-treated groups have smaller changes in the average scores of PC1 component as compared to the control group at the same age, indicating that there is a suppressed progression of the electrophysiological properties when cortical organoids are exposed to methadone.

## Discussion

Previous studies have found that methadone could cause suppression of fetal neurobehavior and alteration in neural maturation in newborns (Wouldes et al., 2004; Jansson et al., 2005, 2011; Walhovd et al., 2012; Monnelly et al., 2018). Furthermore, prenatal exposure to methadone can lead to long-term neurocognitive impairment during child development (Strauss et al., 1976; Rosen and Johnson, 1982; Bauman and Levine, 1986; Davis and Templer, 1988; Bunikowski et al., 1998; Hunt et al., 2008). To better understand the adverse effects of methadone and the underlying mechanisms, we employed human cortical organoids and electrophysiological techniques to investigate the effect of methadone on neuronal function and maturation. We demonstrated that chronic exposure (12-week) to methadone suppressed the maturation of membrane properties and neuronal excitability, which is caused by the attenuation of the function and properties of voltage-dependent ion channels. Since neuronal electrophysiological function is essential for neural maturation in the developing brain (Zhang and Poo, 2001; Zhang and Linden, 2003), we believe that the effects of prolonged methadone exposure on the development and regulation of ion channels contribute to brain maldevelopment in early life. This work, to our knowledge, is the first to use brain organoid model to study the neurodevelopmental impact of prenatal opioid exposure which provides a mechanistic insight of how methadone produces delays in maturation by affecting excitability and ion channel function.

A previous study has shown that methadone concentrations in patients’ plasma are in the low micromolar range with an average level at ∼1 μM (Wolff et al., 1991). A more recent study further demonstrated that the average concentration in 19 placenta samples was around 1,500 ng/g (approximating 5 μM), suggesting that the methadone concentration in the fetus is in low micromolar concentrations as well (De Castro et al., 2011). Therefore, the methadone concentrations (1–10 μM) we studied are clinically relevant.

To understand the molecular basis for the effects of methadone, previous studies have primarily used rodent models in various experimental conditions. Repeated drug injection in rats for a few days showed that methadone could suppress the release of neurotransmitters (McGinty and Ford, 1980; Guo et al., 1990; Ross et al., 2015). Prenatal exposure to methadone could also disrupt oligodendrocyte maturation and myelination in early developing rat brain (Vestal-Laborde et al., 2014). However, the use of rodent models is problematic for studying the effects of opioid exposure on human brain development. The developmental timing and growth of critical events such as neurogenesis, gliogenesis, synaptogenesis, and myelination are vastly different between humans and rodents (Semple et al., 2013; Hauser and Knapp, 2017). The fast neurodevelopment (a few days) of key events in rodents does not make the use of them as an optimal model to be translated into humans. Unlike rodents or two-dimensional (2D) neural culturing method, human 3D brain organoid development takes a prolonged period of time (up to 10 months) which resembles the time course of human fetal brain (Trujillo et al., 2019).

It has been shown that neural excitability and connections can be affected by opioids such as morphine and enkephalin (Hutchinson et al., 2011; Smith et al., 2012; Winters et al., 2017). Regarding methadone, however, there has been no known significant effect on neuronal excitability at clinically relevant doses (≤10 μM). At much higher concentrations (≥100 μM), data exist showing that methadone acutely inhibited neuronal Na^+^ current and excitability (Stoetzer et al., 2015; Yao et al., 2020). In this work, we took advantage of the organoid model to study the effect of methadone for a long period (i.e., a few months). We found that long-term methadone exposure can significantly suppress neuronal excitability by altering the densities and properties of neuronal ion channels. Since 2D neural cultures cannot be maintained for a long-term drug treatment, our work, as an example, showed a critical advantage of using 3D brain organoid to study normal neurodevelopmental maturation and disorders in early life.

To perform electrophysiological recordings, the cortical organoids were plated in the dish, the same condition as for Multi-Electrode Array (MEA) recordings in our recent work (Trujillo et al., 2019). The combination of electrophysiology and immunocytochemistry confirmed the nature of the specific types of neuron and glial cells recorded at the edge of cortical organoids, as previous studies have shown, namely that the vast majority of cells in cortical organoids are glutamatergic neurons and GFAP-immunoreactive astrocytes (Trujillo et al., 2019). It has also been shown that more “maturing” neurons tend to be located in the outer layer of the organoids while other non-functional cells such as neural progenitor cells are prone to be inside the organoids (Trujillo et al., 2019). Indeed, our patch-clamp recordings showed that the cells at the edge of the organoids are mostly functional neurons. An alternative approach is the use of brain organoid slices to perform patch-clamp recordings (Birey et al., 2017; Yoon et al., 2019). Although the organoid slice allows direct recordings of cells in the organoids, the slice cutting process causes severe cell damage, as it has been shown in the brain slice preparation (Aitken et al., 1995; Schurr et al., 1995).

The variability of human brain organoid has been a major challenge in the field (Qian et al., 2019; Velasco et al., 2019; Yoon et al., 2019). In this work, we have used multiple approaches to minimize the potential variability. First, we chose the guided approach to generate cortical organoids, which has been shown to produce lower variability due to a less cell heterogeneity as compared to the whole-brain organoids (Quadrato et al., 2017; Yoon et al., 2019). Second, three iPSC lines used in this work have similar background, including the same tissue source (fibroblasts), reprogramming method (retrovirus virus), culture media (mTSER1) and passage number. Furthermore, we performed parallel electrophysiological recordings between control and methadone-treated groups, with consistent cell locations (<200 μm from the edge of organoids). Indeed, our result in Figure 2 showed that, both passive and active membrane properties are consistent among cortical organoids from three healthy subjects.

Major changes occur in both passive and active membrane properties during early neurodevelopment. Immature neurons have low *C*_*m*_, high *R*_*in*_, depolarized *V*_*rest*_, and low excitability (Luhmann et al., 2000, 2003). During development, neurons undergo dramatic changes in membrane properties and excitability, continuously acquiring their adult phenotype (Rimmer and Harper, 2006; Dufour et al., 2014). Indeed, we found developmental changes of membrane properties in the cortical organoids from 12- till 24-week-old, including increasing *C*_*m*_, decreasing *R*_*in*_, and more negative *V*_*rest*_ (control group, Figure 3 and Table 1). During the same period, we found a significant increase in neuronal excitability, as characterized by changes in evoked AP firing, and single AP properties (Figure 4 and Table 1). In addition, our voltage-clamp recordings revealed a large increase on current sizes and gating properties of voltage-dependent Na^+^ channels and K^+^ channels (Figures 5, 6). In particular, the maturation of AP firing patterns is greatly attributed by the positive shift of voltage dependence of inactivation of neuronal Na^+^ channels, which significantly increases the channel availability (Figure 5). Our recent study using MEA recordings demonstrated a developmental increase of neural network activities in cortical organoids (Trujillo et al., 2019). The high degree of neuronal maturation at both cellular and population levels in cortical organoids resembles the functional development of human fetal brain, suggesting a critical advantage of using such model for neurological studies as compared to 2D neural culture systems or animal models.

We have used voltage-clamp techniques to investigate three types of ionic current components (*I*_*Na*_, *I*_*KD*_ and *I*_*KA*_) that are the electrical basis for AP firing and abundantly expressed in cortical neurons. *I*_*Na*_ is responsible for the initiation of AP depolarization while both *I*_*KD*_ and *I*_*KA*_ have major contributions for AP repolarization. *I*_*KD*_ component provides the basis of AP repolarization while *I*_*KA*_ component regulates the AP firing timing (Coetzee et al., 1999; Misonou et al., 2005; Noh et al., 2019). Interestingly, long-term methadone exposure suppresses the *I*_*Na*_, *I*_*KD*_ components whereas *I*_*KA*_ component is not changed, indicating a specificity of methadone’s action. The methadone-induced reductions of *I*_*Na*_ and *I*_*KD*_ components indicate a weaker driving force for AP depolarization and repolarization, respectively. In addition, the shift of voltage-dependent activation and inactivation of Na^+^ channels also contributes to the weakened driving force for initiating AP firing. Methadone affects the dynamic interaction between *I*_*KD*_ and *I*_*Na*_. After the repolarization of the first AP, the smaller negative membrane potential (due to decreased *I*_*KD*_) in methadone-treated neurons will induce less availability in Na^+^ channels (more inactivation) for the following AP firing. Therefore, these changes in ion channel activities explain how methadone induces the reduction of AP firing frequency and single AP properties. We also characterized the GFAP-positive astrocytes that express *I*_*Kir*_ in cortical organoids (Figure 1). Since astrocytes can modulate neuronal excitability through Kir-mediated K^+^ spatial buffering (Bellot-Saez et al., 2017; Wu et al., 2018), future studies will be needed to determine whether methadone or other opioids affect the development of glial properties.

In summary, we have used human cortical organoids to investigate the longitudinal effect of methadone exposure on early brain development. Methadone at clinical doses suppresses neuronal function and maturation through the attenuation of densities and biophysical properties of neuronal Na^+^ and K^+^ channels. Since intrinsic neuronal excitability is essential for regulating brain development, the decrease in neuronal function via changes in ion channel properties is, we believe, an important mechanism underlying the suppression in the maturation of neuronal development. Our findings represent the first study showing that there is an electrophysiologic basis for the methadone-induced suppression in neuronal development, which will assist us to potentially develop novel therapeutic targets for the treatment of neurological deficits seen in patients prenatally exposed to opioids.

## Data Availability Statement

The original contributions presented in the study are included in the article/supplementary material, further inquiries can be directed to the corresponding authors.

## Author Contributions

WW performed the electrophysiological experiments and analyzed the data. WW, HY, and HZ performed the immunocytochemistry experiments. ID, PN, HZ, JW, and CT provided reagents and cortical organoids. WW and GH designed the study and wrote the manuscript. HY and AM gave conceptual advice and commented on the manuscript at all stages. All authors contributed to the article and approved the submitted version.

## Conflict of Interest

The authors declare that the research was conducted in the absence of any commercial or financial relationships that could be construed as a potential conflict of interest.

## Abbreviations

iPSCs: induced pluripotent stem cells
AP: action potential
*C*_*m*_: cell capacitance
R_*in*_: input resistance
V_*rest*_: resting membrane potential
I_*Na*_: Na^+^ current
I_*K*_: K^+^ current
I_*KA*_: A-type transient K^+^ current
I_*KD*_: delayed rectifier K^+^ current.
